# Point contact Andreev reflection studies of a non-centro symmetric superconductor Re_6_Zr

**DOI:** 10.1038/s41598-019-39160-y

**Published:** 2019-02-21

**Authors:** Pradnya Parab, Deepak Singh, Santosh Haram, R. P. Singh, Sangita Bose

**Affiliations:** 1 School of Physical Sciences, UM-DAE Center for Excellence in Basic Sciences, University of Mumbai, Kalina, Santacruz (East), Mumbai, 400098 India; 2 National Centre for Nanoscience & Nanotechnology, University of Mumbai, Kalina, Santacruz (East), Mumbai, 400098 India; 3 Department of Physics, Indian Institute of Science Education and Research Bhopal, Bhopal Bypass Road, Bhauri, Bhopal, 462066 Madhya Pradesh India

## Abstract

Re_6_Zr, a non-centrosymmetric superconductor is an interesting system as recent experimental evidence suggests that the superconducting state breaks time reversal symmetry. This implies a mixing of spin singlet-triplet states leading to a complex order parameter in this system. Here, we report point contact Andreev Reflection (PCAR) measurements on a single crystal of Re_6_Zr (superconducting transition temperature (T_c_) = 6.78 K). We observe multiple gap features in the PCAR spectra which depends on the type of tip and contact. Spectral features appear at voltages 1.0 ± 0.1 mV, 0.75 ± 0.05 mV and 0.45 ± 0.1 mV suggesting that there are at least more than one band contributing to superconductivity. However, strong surface inter-band scattering is possibly responsible for the uncertainty in observing them together distinctly in a single contact in the PCAR measurements. Interestingly, the bulk gap (Δ  = 1.95k_B_T_c_ = 1.1 meV) is occasionally observed in PCAR spectra, mostly with ferromagnetic tips. The gap features associated with the other two smaller gaps disappear at the bulk T_c_. In addition, no anisotropy in the upper critical field was observed. Our results suggest an unconventional superconducting order in this compound: Multiband singlet states dominated by inter-band pairing which break the time reversal symmetry or singlet mixed with triplet states.

## Introduction

In recent times, non-centrosymmetric superconductors (NCS) have attracted considerable interest owing to the complex nature of superconductivity in these materials^1^. In conventional superconductors, where the crystal structure possesses a center of inversion symmetry the superconducting order parameter (OP) is characterized by a distinct parity corresponding to either a spin-singlet or spin-triplet pairing. However, when the crystal structure lacks a point of inversion symmetry, parity and hence spin is no longer a good quantum number and this classification is no longer adequate. In the absence of inversion symmetry, the antisymmetric spin -orbit coupling (ASOC) lifts the degeneracy between the Bloch states with same crystal momentum, ***k***, but opposite spins, giving rise to two helicity bands where the Fermion spins are pinned along specific directions with respect to ***k***. When the ASOC is large enough that the pairing only occurs between electrons in the same helicity band, the OP can be a mixture of spin-singlet spin-triplet state. When the spin-triplet component is large a NCS can exhibit unconventional properties, such as the upper critical field exceeding the Pauli limit and nodes developing in the superconducting energy gap^1^.

Despite several theoretical predictions, experimental evidence of singlet-triplet mixing in NCS have been surprisingly few. Among NCS that are superconductor at ambient pressure, evidence of nodes in the superconducting energy gap has been obtained for Li_2_Pt_3_B^2,3^, Y_2_C_3_^4^ and CePt_3_Si^5,6^, though in the last one study of parity broken superconducting state is complicated by coexistence of antiferromagnetic order. Yet many other compounds, such as BiPd^7–10^, Nb_0.18_Re_0.82_^11,12^, Re_3_W^13^, PbTaSe_2_^14^ exhibit fully open superconducting gap while some of them show evidence for multiband superconductivity^11,12^. Recently, the superconducting state of the NCS, Re_6_Zr with the α-Mn cubic structure^15^ has called for particular attention. While specific heat, penetration depth and nuclear quadrupole resonance (NQR) data are consistent with a fully gapped state, the upper critical field (H_c2_) is very close to the Pauli limit^16–18^. More importantly, muon spin rotation (μSR) measurements performed on polycrystalline samples reveal that the superconducting state breaks time reversal symmetry (TRS), which the authors suggested as evidence for strong spin singlet- spin triplet mixing^19^. Since singlet triplet mixing is expected to give a rise to a strongly anisotropic gap function, it is important to obtain detailed information of the superconducting gap symmetry on high quality single crystals.

Point contact Andreev reflection (PCAR) spectroscopy^20^ is a powerful tool to investigate the superconducting gap structure in superconductors. In this technique, a ballistic contact is established by bringing a sharp normal metal tip in contact with the surface of the superconductor. The dependence of the differential conductance (*G(V)* = *dI/dV*) as a function bias voltage (*V*) of such a contact is sensitive to the magnitude and symmetry of the superconducting gap function. Consequently, *G(V)*-*V* spectra provides valuable insight on the gap symmetry and its temperature evolution in unconventional superconductors.

## Results

### Sample Characterization

**Figure 1:**
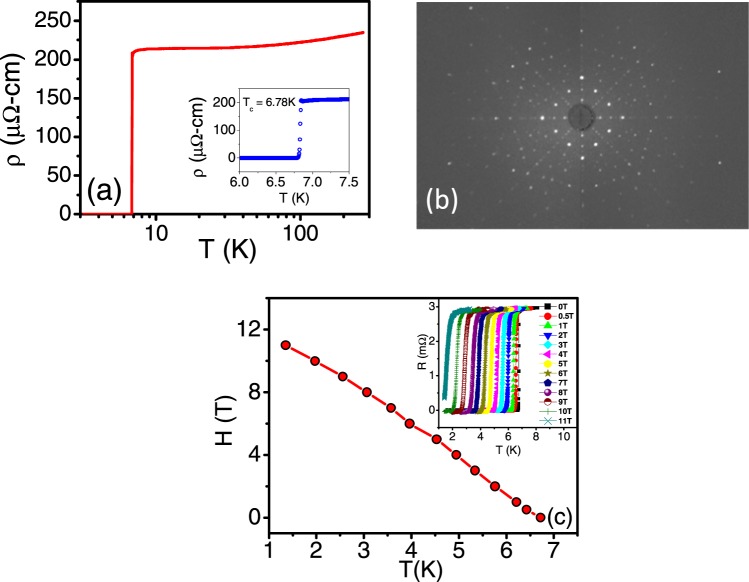
(**a**) Resistivity (ρ) vs Temperature (T) for Re_6_Zr single crystal in zero field. The inset shows the expansion of ρ-T showing the superconducting transition at T_c_ ~ 6.78 K. (**b**) X-ray Laue diffraction pattern of the crystal along the principal axis of the crystal. (**c**) Temperature dependence of H_c2_. The inset shows R-T plot at various magnetic fields.

The Laue diffraction measurement performed on the single crystal used for the present study reveals sharp Bragg spots consistent with growth along the principal axis of the crystal (Fig. 1(b)). XRD data (see Fig. S1 of Supplementary Information) along with the Rietveld refinement indicates the crystal was single phase, forming the α-Mn cubic structure. Besides, EDAX confirmed the atomic ratio to be approximately 6:1. Figure 1(a) shows resistivity versus temperature (ρ − T) measurements at zero magnetic field. We observe a sharp superconducting transition with T_c_ ~ 6.78 K and a normal state resistivity ~200 μΩ-cm at 10 K (See Inset of Fig. 1(a)). The mean free path was estimated to be low^21^. Since Re_6_Zr is a very hard material it is difficult to reduce the intrinsic defects such as vacancies, dislocations by annealing. Scattering from these defects is the likely cause of the low mean free paths. The temperature variation of upper critical field (*H*_*c2*_) is determined from the temperature at which the resistance is 90% of the normal state resistance in fixed magnetic fields (Inset of Fig. 1(c) shows the R-T scans in different magnetic fields). At 1.4 K, *H*_*c2*_ ~ 11 T (see Fig. 1(c)) consistent with earlier reports on polycrystalline samples^19^ as well as single crystals^21^.

### Point Contact Andreev Reflection (PCAR) studies

**Figure 2:**
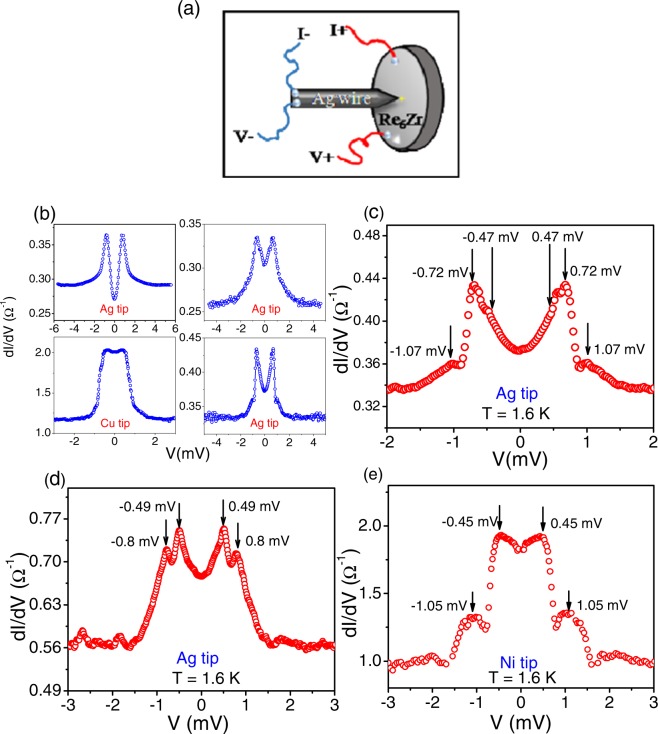
(**a**) Schematic of the point contact between Re_6_Zr sample and a non-superconducting tip. (**b**–**e**) Representative PCAR (*G(V)* (dI/dV) vs *V)* spectra recorded below 2 K on the [100] surface of Re_6_Zr single crystal using normal metal/ferromagnetic tips. (**b**) PCAR spectra with different tips and for different contacts with no dips at higher bias signifying that there is no heating at the contacts. (**c**) PCAR spectra showing three gap features at voltages V_1_ = ±0.40 mV, V_2_ = ±0.72 mV and. V_3_ = ±1.07 mV (**d**) Two gap features are seen in the spectra at voltages V_1_ = ±0.49 mV and V_2_ =  ± 0.80 mV (**e**) Two gap features are seen in the spectra at voltages V_1_ = ±0.45 mV and V_3_ = ±1.05 mV.

PCAR studies were carried out on the Re_6_Zr single crystal with different normal metal and ferromagnetic tips. To obtain spectroscopic information from PCAR measurements one has to ensure that the point contact diameter (*d*) is in the ballistic or the diffusive regime, i.e. *d* < *l*_*in*_, where *l*_*in*_ is the inelastic mean free path^22^. The size of the point contact is often estimated from the contact resistance using the Sharvin formula. However, this method can be misleading since the microscopic structure of the contacts (such as the existence of multiple parallel contacts) is difficult to know. On the other hand, it has been shown that diagnostics of the spectra can be used to determine if the contacts are in the ballistic or diffusive regime. When the contact is in the thermal regime (*d* >> *l*_*in*_) it has been shown that the spectra exhibit pronounced dips at high bias related to the critical current of the superconductor^23^. Spectra showing pronounced dips at high bias were thus discarded for quantitative analysis (Fig. 2(b) shows some representative spectra analyzed in this work upto high bias showing no features related to heating). For most contacts, the contact resistance, *R*_*c*_ was in the range of 0.5 to 5 ohm which would give the contact diameter from the Sharvin resistance formula as 10–50 nm^22,24^. Though we can completely rule out the contacts reported in this work to be in thermal regime based on the spectra obtained, there could be some contacts in the diffusive regime as well (a consequence of the low mean free paths in the sample which results in broadened PCAR spectra) from which we can still get energy resolved information^25^. In Fig. 2(c–e) we show representative *G(V)*-*V* spectra for three different contacts recorded at temperatures below 2 K. All the spectra display multiple gap features. For the spectrum shown in Fig. 2(c) prominent gap features are seen at bias voltage ±0.72 mV (referred to V_2_) and ±1.07 mV(referred to V_3_). In addition symmetric very small humps are seen at voltages ± 0.47 mV (referred to V_1_). Similarly, for the spectrum shown in Fig. 2(d) symmetric peaks are observed at voltages ±0.80 mV and ±0.49 mV and for the spectrum in Fig. 2(e) at voltages ±1.05 mV and ±0.45 mV. This clearly indicates that multiple gaps contribute to superconductivity. To probe this further, more statistics was obtained by repeating the measurements with different contacts and also different tips. (Some of the spectra are shown in Fig. S2 of the Supplementary Information). Interestingly, for some contacts only the gap feature at V_1_ ∼ 0.40 mV was seen distinctly (Fig. S2(a,b)). For some only the gap feature at V_2_ ∼ 0.7 mV was seen (Fig. S2(c,d)) while for some other contacts features at both V_1_ = ±0.4 mV and V_2_ = ±0.7 mV were seen together (Fig. S2(e,f)). The gap feature at V_3_ = ±1.05 mV was observed very rarely (Fig. S2(g,h)) and mostly with ferromagnetic tips like Ni and Fe indicating it to be direction dependent and spin sensitive. This feature corresponds to the bulk gap (Δ  = 1.95k_B_T_c_ = 1.1 meV) in Re_6_Zr^19^. The observation of the multiple gap features in Re_6_Zr indicates that more than one energy band contributes to superconductivity, a situation similar to multi-band superconductivity observed in MgB_2_^20^ or other NCS systems like LaNiC_2_^26^, BiPd^7^, Nb_0.18_Re_0.82_^7^ etc.

**Figure 3:**
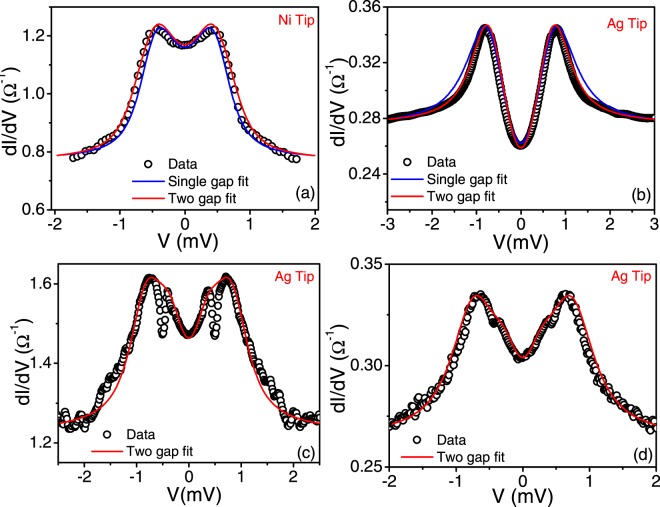
Representative PCAR spectra (open circles) along with their fits (solid lines) with the BTK or modified BTK model (see text). (**a**) Gap feature fitted with a single gap (Blue solid line with best fit parameters of Δ = 0.53 meV, z = 0.33, Γ = 0.03 meV) and two gaps (Red solid line with best fit parameters of Δ_1_ = 0.50 meV, z_1_ = 0.325, Γ_1_ = 0.02 meV, Δ_2_ = 0.72 meV, z_2_ = 0.325, Γ_2_ = 0.02 meV). (**b**) Gap feature fitted with a single gap (Blue solid line with best fit parameters of Δ = 0.74 meV, z = 0.695, Γ = 0.15 meV) and two gaps (Red solid line with best fit parameters of Δ_1_ = 0.39 meV, z_1_ = 0.74, Γ_1_ = 0.15 meV, Δ_2_ = 0.74 meV, z_2_ = 0.74, Γ_2_ = 0.035 meV). The two gap fit improves the fit marginally at high bias.(**c**) Multiple gap features fitted with two-gaps with best fit parameters as Δ_1_ = 0.39 meV, z_1_ = 0.58, Γ_1_ = 0.032 meV, Δ_2_ = 0.86 meV, z_2_ = 0.47, Γ_2_ = 0.003 meV (Red solid line). (**d**) Multiple gap features fitted with two-gaps with best fit parameters as Δ_1_ = 0.35 meV, z_1_ = 0.43, Γ_1_ = 0.012 meV, Δ_2_ = 0.74 meV, z_2_ = 0.43, Γ_2_ = 0.1 meV (Red solid line).

To obtain quantitative information we fit the spectra showing distinctly a single gap feature with the Blonder-Tinkham-Kalpwijk^27^ (BTK) model with an isotropic gap according to which the current versus voltage characteristics of a N/S point contact is given by:

1 $$I(V)\propto N(0){v}_{F}{\int }_{-\infty }^{\infty }[f(E-eV,T)-f(E,T)][1+A(E)-B(E)]dE$$

where f(E) is the Fermi-Dirac distribution function, N(0) is the density of states of the normal metal at Fermi level and v_F_ is the Fermi velocity of the normal metal. A(E) is the Andreev reflection probability and B(E) is the normal reflection probability. From Eq. 1, G(V) (dI/dV) versus voltage (V) or the PCAR spectra can be simulated. The broadening arising from the finite lifetime (τ) of the superconducting quasi-particle can be incorporated in the BTK model by replacing Ε → Ε + iΓ which modifies the expression of A(E) and B(E)^28^ which now become a function of Γ also. The parameter, ${\rm{\Gamma }}=\frac{h}{\tau }$ phenomenologically accounts for the finite lifetime of the superconducting quasiparticle^29^, provided Γ is much smaller than the characteristic superconducting energy scale (Δ). Γ in practice incorporates all non-thermal sources of broadening, e.g. distribution of superconducting energy gaps, instrumental broadening etc. We use Δ, the dimensionless barrier potential at the interface (z) and Γ as fitting parameters to fit the experimental data with the BTK model. We observe that the fits broadly capture the shape of the spectra giving a value of the gaps as Δ_1_ = 0.53 meV, corresponding to V_1_ (see Fig. 3(a)) and Δ_2_ = 0.74 meV corresponding to V_2_ (Fig. 3(b)). For the spectrum shown in Fig. 3(b) we observe that the fit deviates significantly at bias voltages above the coherence peak. In order to fit the spectra in Fig. 3(c) and (d) showing two distinct gap features we use a two-band BTK model^30^, where, current (*I)*, and hence *G* is a weighted sum from two transport channels [*G*_*1*_*(V)* and *G*_*2*_*(V)*], arising from two bands in the superconductor. In this model the normalized conductance *G(V)/G*_*N*_ (*G*_*N*_ = *G(V* >> Δ/*e)*, wher*e e* is the electronic charge) is given by, *G(V)/G*_*N*_ = (1 − *w*)G_1_*(V)/G*_*1N*_ + *wG*_*2*_*(V)/G*_*2N*_. *G*_*1*_*(V)/G*_*1N*_ and *G*_*2*_*(V)/G*_*2N*_ are calculated using the generalized BTK formalism using the relative weight factors of the two gaps (*w*), superconducting energy gaps (Δ_1_ and Δ_2_), the barrier potentials (z_1_ and z_2_), and the broadening parameters (Γ_1_ and Γ_2_) as fitting parameters. We observe that the two-gap model with two isotropic gaps provide a good fit for the spectra shown in Fig. 3(c) and (d). Interestingly, the spectra shown in Fig. 3(a,b) show a marginally better fit with the two band model especially at high bias. From the two gap fits we obtain the two gaps as Δ_1_ ~ 0.40 ± 0.1 meV, and Δ_2_ ~ 0.76 ± 0.10 meV and the weightage (w) of the dominant gap being greater than 0.52. Γ1, Γ2 < 0.15 meV which depends on the contact. We believe that the primary source of spectral broadening here is from interfacial defects which varies from contact to contact. It is also important to note that in none of the spectra we observe any evidence of any zero bias conductance peak which is a characteristic feature associated with the presence of the Andreev bound state expected to be observed for NCS superconductors^1,31^.

**Figure 4:**
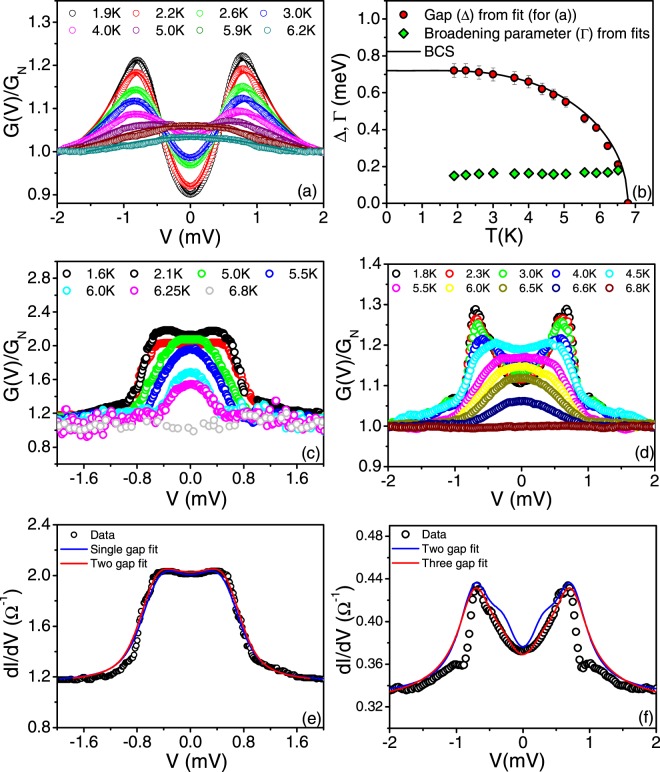
Temperature evolution of the PCAR spectra for (**a**) the contact shown in Fig. 3(b). The solid lines are the fit to the one-gap BTK model. (**b**) Temperature variation of Δ_2_ (Red circles) and Γ (green diamonds) obtained from the fits of data in (**a**). The dashed black lines show the expected BCS temperature variation for the gap (**c**) Temperature evolution of the PCAR spectra for the contact shown in Fig. 3(a). The gap feature disappears at the bulk T_c_ = 6.8 K. (**d**) Temperature dependence of the PCAR spectra for the contact shown in Fig. 2(c). Gap features disappear at the bulk T_c_ = 6.8 K.(**e**) Fit of the data shown in (**c**) at the lowest temperature using the one band BTK fit (blue line, with best fit parameters Δ = 0.60 meV, z = 0.18, Γ = 1e-5 meV) and two band BTK fit (Red line, with best fit parameters of Δ_1_ = 0.60 meV, z_1_ = 0.18, Γ_1_ = 0.025 meV, Δ_2_ = 0.79 meV, z_2_ = 0.18, Γ_2_ = 0.025 meV). (**f**) Fit of the data shown in Fig. 2(c) using the two band BTK fit (blue line, with best fit parameters of Δ_1_ = 0.33 meV, z_1_ = 0.48, Γ_1_ = 0.05 meV, Δ_2_ = 0.76 meV, z_2_ = 0.58, Γ_2_ = 0.048 meV) and three band BTK fit (Red line, with best fit parameters of Δ_1_ = 0.33 meV, z_1_ = 0.48, Γ_1_ = 0.05 meV, Δ_2_ = 0.76 meV, z_2_ = 0.58, Γ_2_ = 0.048 meV, Δ_3_ = 1.0 meV, z_3_ = 0.25, Γ_3_ = 0.6 meV).

We now investigate the temperature dependence of gaps Δ_1_, Δ_2_ and Δ_3_ by tracking the temperature dependence of the point contact spectra (Fig. 4(a),(c) and (d)). All PCAR spectra become featureless around T = 6.8 K indicating that the largest gap (Δ_3_) closes at the bulk T_c_. The *G(V)*-*V* spectra of Fig. 3(b) showing distinctly the feature corresponding to the gap Δ_2_ was fitted with the single gap BTK model at all temperatures (See Fig. 4(a)). As seen from Fig. 3(b), the fit with two gaps does not improve the quality of fit substantially at the lowest temperature and besides the value of Δ_2_ obtained from both fits remains unchanged. The temperature variation of Δ_2_ obtained from the fits is shown in Fig. 4(b). (We also plot the temperature variation of Γ obtained from the fits and observe that it is much lower than Δ_2_). Δ_2_ follows the temperature dependence from Bardeen-Cooper-Schriffer (BCS) theory and this gap also closes at T_c_. (We confirmed this by analyzing another contact showing predominantly the Δ_2_ gap feature distinctly, see Fig. S3 of Supplementary Information). The *G(V)-V* spectra showing distinctly the feature corresponding to the gap Δ_1_ for different temperatures are shown in Fig. 4(c). They could not be fitted with the single gap BTK model for all temperatures. Figure 4(e) shows the fit with one gap (Δ_1_) and two gaps (Δ_1_ and Δ_2_) of the spectra at the lowest temperature. No substantial improvement is visible between the two fits. At higher temperatures, we believe that the small feature at V_3_ corresponding to Δ_3_ starts to dominate making it increasingly difficult to fit the spectra. Figure 4(d) shows the temperature dependence of the PCAR spectra where all the three gap features are distinctly resolved at the lowest temperature. We tried fitting the spectra at T = 1.6 K with two and three band BTK models where each gap is expected to be isotropic. However, neither gave a reasonable fit and the gap feature at V_3_ = 1.05 mV was difficult to reproduce (see Fig. 4(f)). Thus, any spectra showing features corresponding to Δ_3_ was difficult to fit at all temperatures using the isotropic BTK model.

**Figure 5:**
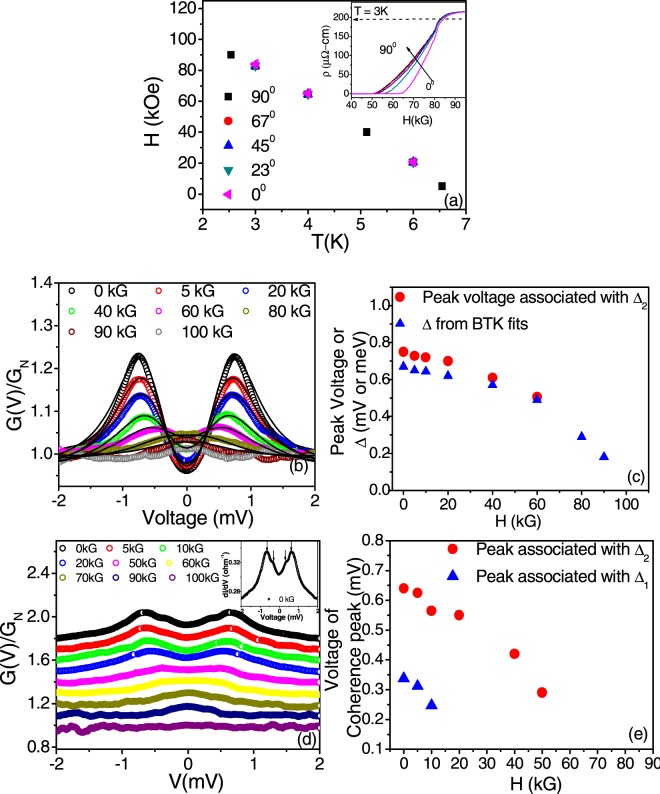
(**a**) Phase diagram (H_c2_ vs T) for Re_6_Zr. The H_c2_ was obtained from magneto-resistance measurement at different temperatures and for different angles (shown by different symbols) between the current direction and the magnetic field. The inset shows the ρ vs H at 3 K for different angles. No anisotropy in H_c2_ was observed. (**b**) and (**d**) Magnetic field evolution of the normalized PCAR spectra for two different contacts. In (**b**), the symbols are the experimental data and the solid lines are the BTK fits. The inset in (d) shows the PCAR spectra at a magnetic field of 0 kG showing the peak features distinctly associated with the two gaps Δ_1_ and Δ_2_ (**c**) Variation of the voltage of the coherence peaks (red solid circles) and energy gap (Δ) obtained from the BTK fits (blue triangles) with magnetic field (H) for the data shown in (**b**). (**e**) Variation of the voltage peaks associated with the gaps Δ_1_ and Δ_2_ with magnetic field of the data shown in (**d**).

To reconcile with the observation of different gaps from the PCAR spectra, it is important to check if there is any anisotropy in the superconducting properties for the NCS superconductor Re_6_Zr. We studied the angular dependence of the upper critical field (H_c2_) though resistivity (ρ) measurements in magnetic field (H) for different temperatures below T_c_. The ρ *vs* H plot at 3 K for different angles of the sample transport current to the magnetic field is shown in the inset of Fig. 5(a). The sample was rotated with respect to the axis of the magnetic field. H_c2_ was taken as the field at which the resistivity is 90% of the normal state value (shown by the dashed line in the inset). The phase diagram for angles between 0° to 90° are obtained from this measurement and is shown in Fig. 5(a). No substantial anisotropy was observed in H_c2_. Furthermore, magnetic field dependence of Andreev spectroscopy was carried out for a few contacts at T = 2 K upto 11 Tesla (Representative spectra are shown in Fig. 5(b) and (d)). PCAR spectra became featureless at 11 T consistent with the measured H_c2_ value for Re_6_Zr from resistivity measurements. For spectra showing only one gap feature distinctly, the spectra could be fitted with the BTK model and Δ could be extracted from the fits. Δ decreased with H and appear to close at H_c2_ (See Fig. 5(c)) (Note: The voltage of the coherence peaks of the PCAR spectra show the same variation with H as Δ (see blue triangles in Fig. 5(c)). For spectra which showed two or three gap features at H = 0, on applying magnetic field, the features smeared out (See Fig. 5(d)) making it difficult to resolve the peaks associated with the individual gaps at higher fields. To see how the two coherence peaks associated with the two gaps, Δ_1_ and Δ_2_ shown in Fig. 5(d) vary with magnetic field, we plot the voltage corresponding to the coherence peaks in the spectra as a function of H in Fig. 5(e). Due to the broadening caused by the strong pair breaking effect of the magnetic field, it is difficult to make out from the spectra if magnetic field causes any collapse of the double-gap state.

## Discussions

We now discuss the implication of these results. First, we explore the possibility of singlet-triplet mixing as conjectured from the observation of TRS breaking in μSR measurements in Re_6_Zr^19^. For a NCS, ASOC leads to a term of the form α*g*(***k***)·σ in the Hamiltonian, where α is the spin-orbit coupling constant, σ is the Pauli matrices, and the vector g(***k***), representing the orbital direction, obeys the antisymmetric property such that *g*(***k***) = −*g*(−***k***). The ASOC breaks the spin degeneracy, which leads to two bands characterized by ± helicities for which the spin eigen states are either parallel or antiparallel to *g*(***k***). The superconducting energy gap on both these bands will have singlet and triplet components. The system will therefore have two gaps defined on each of ASOC split bands, ${{\rm{\Delta }}}_{\pm }(\bar{k})={{\rm{\Delta }}}_{s}\pm {{\rm{\Delta }}}_{t}(\bar{k})$, where Δ_*s*_ and Δ_*t*_(***k***) are the spin singlet and spin triplet component of the gap function. Since Δ_*t*_(***k***) is strongly anisotropic and changes sign depending on ***k*** direction, a significant Δ_*t*_(***k***) component implies that both ${{\rm{\Delta }}}_{\pm }(\bar{k})$ would be strongly anisotropic with a large distribution of gap amplitude over the Fermi surface. Re_6_Zr has a cubic symmetry and the crystal on which measurements were done was oriented along [100] direction. Since the sample does not cleave easily along any other plane, it was not possible to do directional PCAR on Re_6_Zr to check any anisotropy effect. However, by changing contacts through pressure it was possible to microscopically probe different **k** directions. In our experiments, we have observed that the gap feature at V_3_ = 1.05 ± 0.05 meV was very sensitive to the contact as well as tip. It was observed more frequently with ferromagnetic tips indicating the presence of spin polarization. Moreover, in the spectra it was visible, its weight seemed to be low (a small feature) and it could not be fitted with the isotropic gap BTK model (see Fig. 4(e)). These observations could indicate that this gap feature is associated with the triplet gap and we conclude that in this scenario spin-singlet spin-triplet mixing if at all present is very small. This is also consistent with our observation of almost no anisotropy in H_c2_. The small spin singlet-triplet mixing is also expected from the small band splitting due to ASOC (~30 meV)^16^ calculated for this compound which is comparable to the ASOC spin splitting in Li_2_Pd_3_B where a fully gapped isotropic order parameter has been inferred from penetration depth measurements^32^. (In the isostructural NSC Li_2_Pt_3_B where penetration depth measurements provide evidence of large anisotropy and possible nodes in the gap function, the ASOC band splitting is ~200 meV). On the other hand, the spin singlet component of the gap function has contribution from two isotropic energy gaps (Δ_1_ and Δ_2_) which are present on two different Fermi surface pockets. The uncertainty in their observations in different spectra with different tips and contacts implies that there is strong inter-band scattering. It is also possible that these two gaps are surface sensitive and hence do not show up in bulk measurements like specific heat. Thus, the possible scenario which emerges is that there is unconventional superconductivity in Re_6_Zr with very weak spin singlet and spin triplet mixing and presence of multiband superconductivity.

A second possibility of the interpretation of our data could be that all the three gaps are completely isotropic (as evidenced from specific heat measurements of a fully gapped state) and Re_6_Zr behaves as a conventional multiband superconductor. It is worthwhile to note that the band structure of Re_6_Zr reveals there are multiple bands crossing the Fermi level and that some of these bands are strongly split by spin-orbit coupling^16^. This is consistent with our observation of having multiple bands contributing to superconductivity. The pertinent question which follows is, how can one reconcile these results with the observation of TRS breaking in μSR measurements? To answer this question two possible scenarios have been suggested where a multiband superconductor can break TRS. The *first scenario* has been proposed for a multiband superconductor with high symmetry^33^ such as the cubic structure of Re_6_Zr. In such a system, TRS can get broken even with conventional s-wave singlet pairing. Under certain conditions, when the Coulomb repulsion between two Fermi surface pockets dominate, the relative phase between one pocket and another will be non-zero. Such a superconducting state will break TRS, allowing antiferromagnetic domains and fractional vortices to appear. A *second scenario* has been proposed in the context of TRS breaking in LaNiC_2_ and LaNiGa_2_^34^. Here pairing occurs between electrons of the same spin but on different orbitals. This gives rise to a novel non-unitary triplet state, where the gap symmetry continues to have even parity. In this case, TRS breaking triplet superconductivity can be realized even when the Fermi surface remains fully gapped. This model of a fully-gapped triplet pairing has also been used to explain the behavior of iron pnictides^35^. Distinguishing between these two scenarios would require a detailed knowledge of the Fermi surface structure properties in this compound.

## Conclusions

In summary, PCAR spectra on a Re_6_Zr single crystal shows three gap features. One of these gap features at a voltage of 1.0 ± 0.1 mV corresponding to the bulk gap (Δ(0) = 1.95k_B_T_c_) as observed in specific heat measurements appear to be tip sensitive and has a low spectral weight. Our results conclusively show that one of the gaps (0.75 ± 0.05 meV) is isotropic. Our measurements suggest that the superconducting state has unconventional pairing. Two possible scenarios can be invoked to understand the results. (1) the bulk gap is spin triplet with a very small spin-singlet-triplet mixing and the isotropic spin singlet gaps are surface sensitive and are associated with different Fermi surface sheets. (2) Re_6_Zr is a multiband superconductor with strong inter-band scattering and a fully gapped isotropic superconducting state. In this model, the TRS is broken at the superconducting transition, due to the presence of non-unitary triplet pairing where pairing occurs between electrons with same spin but on different orbitals. Thus, our experiments reiterate an unusual superconducting state in Re_6_Zr which breaks the time reversal symmetry: Spin singlet-triplet mixing or multiband singlet states driven by inter-band pairing. Validation of these scenario will require further theoretical and experimental studies.

## Methods

In this article, we report PCAR measurements on a high quality single crystal of Re_6_Zr. The single crystal was grown using Czochralski crystal pulling method using a tetra-arc furnace under argon atmosphere, starting from a polycrystalline Re_6_Zr button. Polycrystalline sample of Re_6_Zr was prepared by arc melting stoichiometric quantities of Re (99.99%, Alfa Aesar) and Zr (99.99%, Alfa Aesar) on a water-cooled copper hearth in a high-purity Ar atmosphere also in a tetra-arc furnace. The button was melted several times to ensure phase homogeneity. The observed weight loss during the melting was negligible. The phase purity of the polycrystalline button was checked by powder X-ray diffraction. In the Czochralski growth, a tungsten rod was used as the seed, and the crystal was pulled at the rate of 30–50 mm/h.

For PCAR measurements we establish contacts by mechanically engaging a fine normal metal or ferromagnetic tip on [100] surface of the Re_6_Zr single crystal inside a conventional ^4^He cryostat (Fig. 2(a)). The differential conductance, *G*(*V*), is obtained by numerically differentiating the current versus voltage (*I-V*) characteristics recorded at fixed temperatures and magnetic fields to give the PCAR spectra.

## Supplementary information

Point contact Andreev reflection studies of a non-centro symmetric superconductor Re6Zr

## Data Availability

The datasets generated during and/or analysed during the current study are available from the corresponding author on reasonable request.
