# Supplementary material for: Point contact Andreev reflection studies of a non-centro symmetric superconductor Re6Zr
Source: Sci Rep. 2019 Feb 21;9:2498. doi: 10.1038/s41598-019-39160-y (PMC6385355; doi:10.1038/s41598-019-39160-y)
Supplement: Supplementary file 1 — Point contact Andreev reflection studies of a non-centro symmetric superconductor Re6Zr [file 41598_2019_39160_MOESM1_ESM.docx]

***Supplementary information***

**Point contact Andreev reflection studies of a non-centro symmetric superconductor Re_6_Zr**

Pradnya Parab*^a,b^*, Deepak Singh*^c^*, Santosh Haram*^b^*, R. P. Singh*^c^[[1]](#footnote-1)^^*, Sangita Bose*^a^[[2]](#footnote-2)^^*

*^a^* *School of Physical Sciences*, *UM-DAE Center for Excellence in Basic Sciences, University of Mumbai, Kalina , Santacruz (East), Mumbai 400098, India*

*^b^ National Centre for Nanoscience & Nanotechnology, University of Mumbai, Kalina, Santacruz (East), Mumbai 400098, India*

*^c^* *Department of Physics, Indian Institute of Science Education and Research Bhopal, Bhopal Bypass Road, Bhauri, Bhopal 462066, Madhya Pradesh, India*


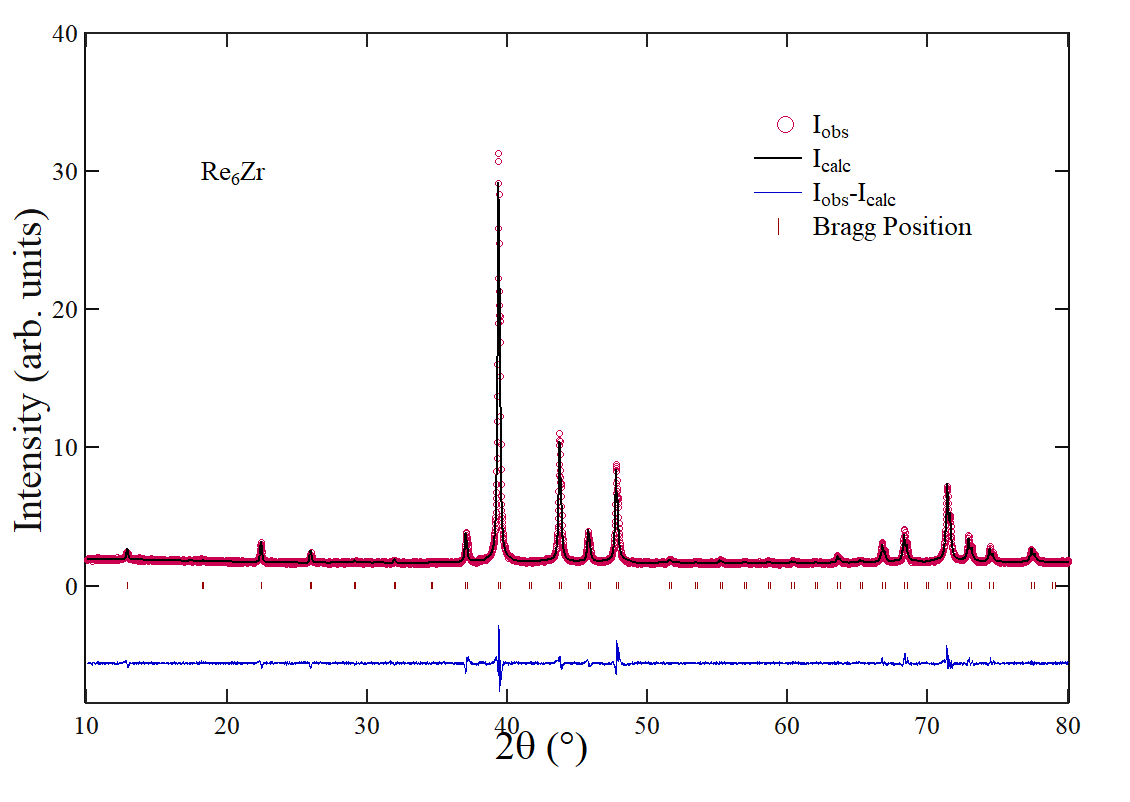


**Figure S1**: XRD of Re_6_Zr along with the Riteveld refinement. No impurity phase was observed.


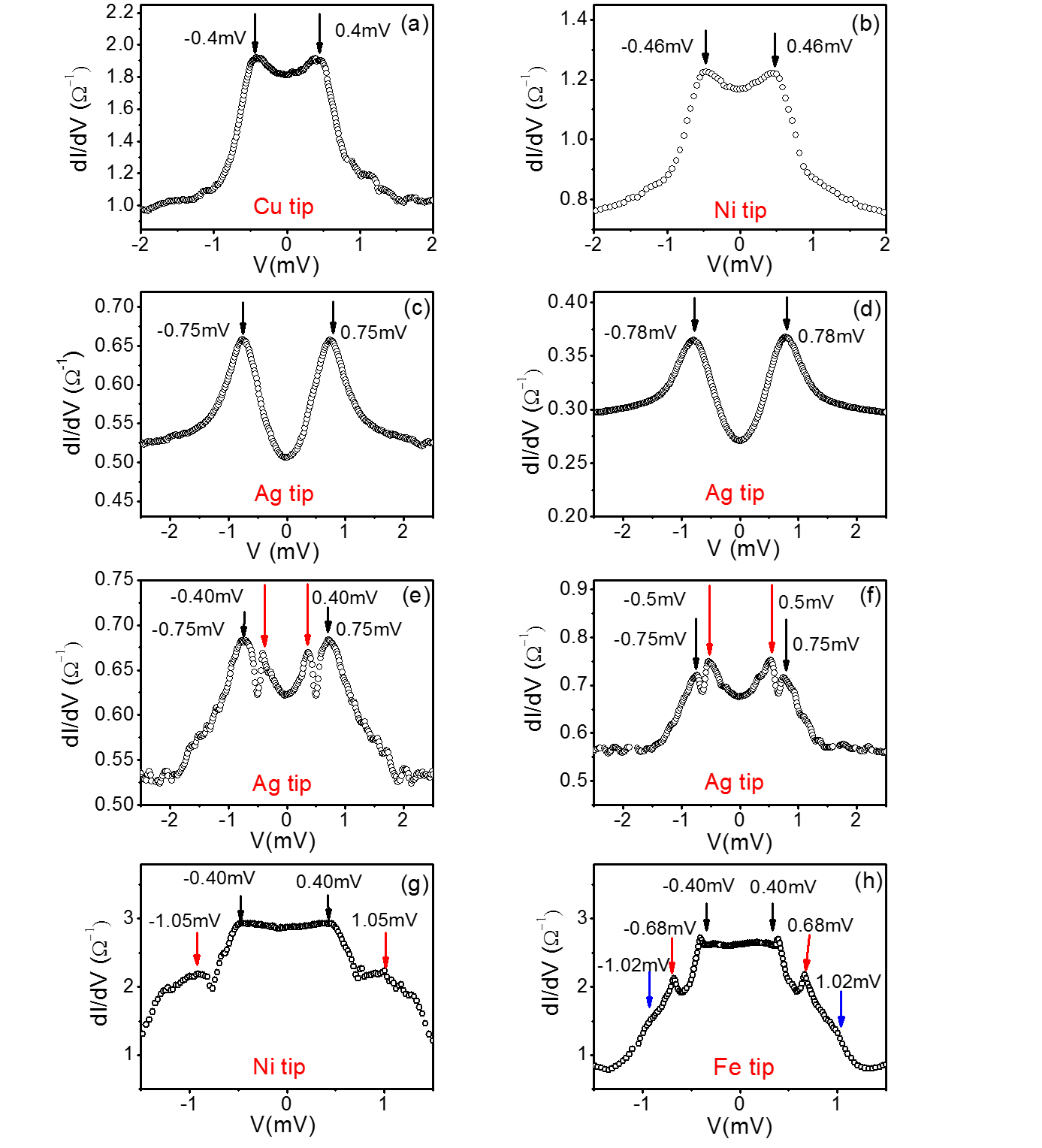


**Figure S2:** Representative PCAR spectra on the [100] surface of Re_6_Zr with different tips and different contacts. Gap features at bias voltage (a – b) ranging between 0.4 to 0.5 mV with Cu and Ni tip respectively. (c – d) ranging between 0.7 to 0.8 mV with Ag tips (e – f) ranging between 0.4 to 0.5 mV and 0.7 – 0.8 mV with Ag tips (g) 0.4 and 1.05 mV with Ni tip and (h) 0.4, 0.68 and 1.02 mV with Fe tip.


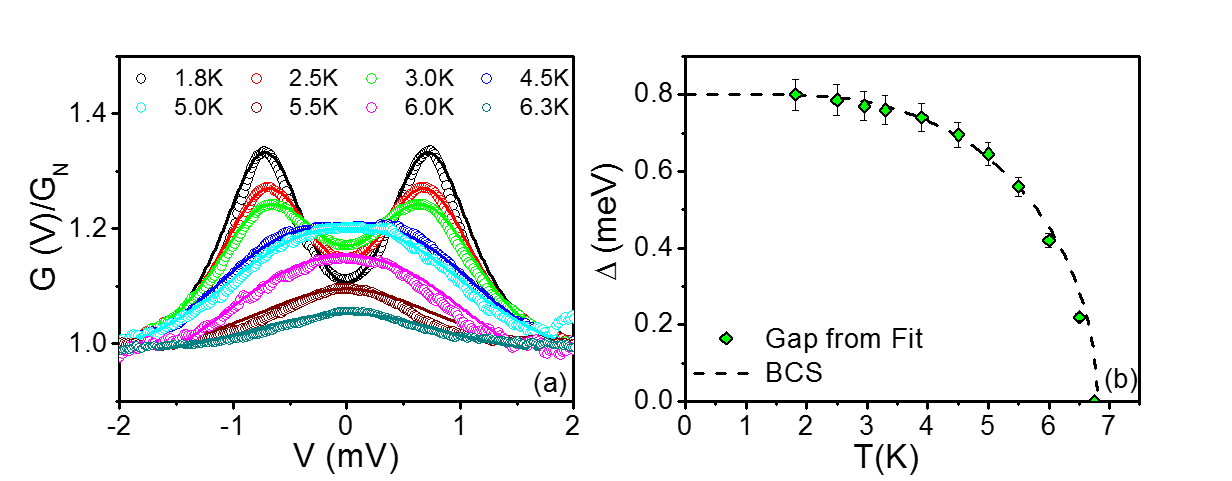


**Figure S3**: Temperature evolution of the PCAR spectra for (a) a contact showing distintly the feature at V_2_ = 0.75 mV. The solid lines are the fit to the one-gap BTK model. (b) Temperature variation of Δ obtained from the fits (Green diamonds). The dashed black lines show the expected BCS temperature for the gaps.

1. [rpsingh@iiserb.ac.in](mailto:rpsingh@iiserb.ac.in) [↑](#footnote-ref-1)
2. [sangita.bose@gmail.com](mailto:sangita.bose@gmail.com) [↑](#footnote-ref-2)
